# Outcome of radioactive iodine therapy in Toxic Nodular Goiter in Pakistan

**DOI:** 10.12669/pjms.345.15244

**Published:** 2018

**Authors:** Tauseef Ahmad, Adeel Khoja, Naveed Haroon Rashid, Muhammad Areeb Ashfaq

**Affiliations:** 1 Dr. Tauseef Ahmad, FCPS, Assistant Professor of Medicine/Endocrinology, Dr. Ziauddin University Hospital, Karachi, Pakistan; 2 Dr. Adeel Khoja, M.Sc (Epidemiology and Biostatistics), Aga Khan University Hospital, Karachi, Pakistan; 3 Dr. Naveed Haroon Rashid, FCPS, Sr. Instructor, Aga Khan University Hospital, Karachi, Pakistan; 4 Muhammad Areeb Ashfaq, Medical Student, Aga Khan University Hospital, Karachi, Pakistan

**Keywords:** Outcome, Radioactive iodine, Single center, Toxic nodular goiter

## Abstract

**Objective:**

To determine the outcome of patients receiving radioactive iodine therapy for toxic nodular goiter coming to Aga Khan University Hospital Karachi.

**Methods:**

A total of 89 patients who visited the outpatient department of Aga Khan University Hospital from January 2010 to August 2017 were recruited for the study. Toxic nodular goiter was diagnosed on the basis of having hot nodule on thyroid scan with low TSH and high FT4/T4. Other demographic and laboratory data were also recorded.

**Results:**

Eighty nine patients with toxic nodular goiter received a dose range from 10 to 30mCi RAI. Six months after RAI, 36.2% became hypothyroid, 38.5% became euthyroid while 25.3% remained hyperthyroid. Thyroid outcome at 3 months were correlating with 6 months results.

**Conclusion:**

Radioactive iodine therapy is a safe and effective way of treating toxic nodular goiter which usually results in cure of hyperthyroidism in majority of patients.

## INTRODUCTION

Toxic thyroid nodule is the second most common cause of hyperthyroidism after graves’ disease.^1^ It is actually a thyroid nodule that becomes autonomous and results in hyperthyroidism. The autonomic stimulation is caused by somatic mutation in thyrotropin or TSH receptors in about 20-80% of toxic nodules and also in some multi-nodular goiter.^2^

Toxic thyroid nodule is different from Graves’ disease as it is not an autoimmune disease and if treated adequately does not remit.^3^ American association of clinical endocrinologist and American thyroid association have recommended radioactive iodine (RAI) or surgical intervention to treat toxic nodular goiter.^4^ Surgical management although can cure this problem but has many anesthesia and surgery related complications.^5^ RAI is an alternative to surgery and is effective in treating benign nodular goiter.^6^

Treatment with a single dose of RAI usually achieve success in 85-100% of patients with toxic nodular goiter and also shrink the size of the goiter up to 40%^7^,^8^ RAI therapy is dependent on iodine uptake by the thyroid tissue and its reduce uptake leads to the treatment being inefficient and more difficult.^9^

RAI therapy in toxic thyroid nodules unlike Graves’ disease, where majority of the patient becomes hypothyroid, usually result in patient becoming euthyroid, as the toxic nodule usually takes majority of radiation. Our study reported the outcome of RAI on toxic nodular goiter and also compares the accuracy of thyroid function test (TFTs) after 3 months of RAI as compared to six months.

## METHODS

A total of 89 patients with toxic nodular goiter who visited our outpatient department at Aga Khan University Hospital from January 2010 to August 2017 were included in our study. Toxic nodular goiter was diagnosed on the basis of suppressed TSH, increase serum FT4/T4 levels and hot nodule on thyroid scan. Patient who were diagnosed to have thyroiditis or Graves’ disease were excluded from the study. All patients who had previous thyroid surgery, neck radiation or had prior RAI treatment were also excluded from the study. All those patients who were lost to follow-up were also omitted from the study.

In all these patients, all the clinical information like age, sex, height and weight were recorded. Serum TSH and T4/FT4 levels were measured by ELISA technique. Patients who had suppressed TSH levels, increased T4/FT4 levels and had hot nodule on thyroid scan were labeled to have toxic nodular goiter. RAI dose was calculated clinically by consultant nuclear physician on the basis of size of thyroid nodule, thyroid function test and radioactive iodine uptake on thyroid scan. At each follow up visit, data was recorded for serum TSH andT4/FT4 levels after an interval of three and six months.

All of our patients were on antithyroid medication (Carbimazole) prior to RAI as per titration method according to American Thyroid Association(ATA) guidelines^10^ for the last 2-6 months and it was stopped five days before RAI. The thyroid status before RAI was also recorded as hypothyroid, euthyroid or hyperthyroid to access its effect on the post RAI outcome.

Frequencies with percentages were reported for all the categorical variables in the dataset. Variable of Age was converted into categories to give a meaningful interpretation of the data with the outcome. Association of Pre RAI TSH and TSH after six months was done using Chi-Square test statistics (expected cell count was greater than 5) or Fischer-Exact test statistics (expected cell count was less than 5). Association of outcome i.e. TSH after six months with age, gender and radioactive iodine dose was assessed through the same statistical tests as mentioned above. Comparison between three months and six months TSH values were also assessed through the same statistical test. All the analysis was done using Stata version 12.

## RESULTS

From January 2010 to August 2017, 89 patients visited Aga Khan University Hospital for toxic nodular goiter and were given respective radioactive iodine treatment. A dose of 15 and 20mCi of RAI was given to majority of the thyroid patients in our study. At six months follow-up visit, majority of the patients became Euthyroid (38.2%), followed by hypothyroid (37.0%), while 24.7% remained hyperthyroid.

As all of them were on antithyroid drugs before getting RAI so majority of study participants, who were in the category of hyperthyroid, converted to Euthyroid at 6 months after RAI, to be precise 36.5%. Moreover, those falling in the category of Euthyroid, majority of them either remained in the same category or shifted to hypothyroid 6 months after RAI, 43.8% to be precise (equal distribution among the two categories). For study participants falling in the category of hypothyroid; majority of them remained in the category of hypothyroid 6 months after RAI, 50% to be precise. Overall, the results are not statistically significant since the p-value is 0.479 (calculated by Fischer Exact Test) (Table-I).

**Table-I:** Comparison of thyroid status before and 6 months after RAI.

Thyroid Status before RAI (on antithyroid medication) No. (%)	Thyroid Status 6 months after RAI		

	Hyperthyroidism No. (%)	Euthyroidism No. (%)	Hypothyroidism No. (%)
Hyperthyroidism 63(70.7)	19 (30.2)	23 (36.5)	21 (33.3)
Euthyroidism 16(17.9)	2 (12.4)	7 (43.8)	7 (43.8)
Hypothyroidism 10(11.2)	1 (10)	4 (40)	5 (50)

Overall p-value of the Table = 0.479 (Fischer Exact Test)

The variable of age was converted into four meaningful categories, category one included study participants from 28 to 40 years, category two included study participants from 41 to 55 years, category three included study participants from 56 to 70 years, category four included study participants from 71 years and above. For those study participants, falling in the age group of 28 to 40 years, majority of them were hyperthyroid, 38.9% to be precise; study participants falling in the age bracket of 41 to 55 years, majority of them were hypothyroid, 43.8% to be precise; study participants falling in the age bracket of 56 to 70 years, majority of them were Euthyroid and hypothyroid (equal distribution), 37% to be precise; and finally study participants falling in the age bracket of 71 years and above, majority of them were Euthyroid, 50% to be precise. Overall, the results are not statistically significant since the p-value is 0.543 (calculated by Fischer Exact Test) (Table-II).

**Table-II:** Effect of age on thyroid status 6 months after RAI.

Age (Years)	Thyroid Status 6 months after RAI		

	Hyperthyroidism No. (%)	Euthyroidism No. (%)	Hypothyroidism No. (%)
28-40	7 (38.9)	5 (27.8)	6 (33.3)
41-55	5 (15.6)	13 (40.6)	14 (43.8)
56-70	7 (26)	10 (37)	10 (37)
>71	3 (25)	6 (50)	3 (25)

Overall p-value of the Table = 0.543 (Fischer Exact Test)

Majority of males were falling in the category of hypothyroid after 6 months of RAI, 41.7% to be precise, majority of females were falling in the category of Euthyroid after 6 months of RAI, 38.8% to be precise. Overall, the results are not statistically significant since the p-value is 0.769 (calculated by Chi-Square Test).

Overall radioactive iodine dose was significantly associated with the outcome i.e. TFTs after 6 months (p-value of 0.04, calculated by Fischer Exact Test). When a dose of 10mCi of I^131^ was given to the study participants, all remained hyperthyroid after 6 months. When a dose of 15mCi of I^131^ was given to the study participants, 50% became hypothyroid after 6 months, 50.0% to be precise. When a dose of 20mCi of I^131^ was given to the study participants, 55% became Euthyroid after six months, 55% to be precise. When a dose of 25mCi of I^131^ was given to the study participants, 50% became hypothyroid after 6 months,. Finally, when a dose of 30mCi of I^131^ was given to the study participants, 66% became hypothyroid after six months. (Table-III).

**Table-III:** Effect of different doses of RAI on thyroid outcome after 6 months.

RAI dose (mCi)	Thyroid Status after 6 months of RAI		

	Hyperthyroidism No. (%)	Euthyroidism No. (%)	Hypothyroidism No. (%)
10	2 (0)	0 (0)	0 (100)
15	10 (25)	10 (25)	20 (50)
20	9 (23)	21 (55)	8 (21)
25	1 (16.7)	2 (33.3)	3 (50)
30	0 (0)	1 (33)	2 (66)

Overall p-value of the Table = 0.040 (Fischer Exact Test)

Study participants who were hyperthyroid at three months, 50% of them remained hyperthyroid at 6 months after RAI, to be precise 50%. Moreover, those who were Euthyroid at three months, 70.9% of them remained Euthyroid 6 months after RAI. For study participants, who were hypothyroid at three months, 67.7% remained hypothyroid 6 months after RAI. The test statistics (Fischer Exact Test) p-value is < 0.001 and it is highly significant. This means, that the results of TFTs at three months do predict the accuracy of the results of TFTs at six months. The results of all the three different categories are accurate and we can predict/conclude that the results at three months and at 6 months are somewhat similar (Table-IV).

**Table-IV:** Comparison between 3^rd^ month and 6^th^ month thyroid function test after RAI.

Thyroid Status after 3 months of RAI	Thyroid Status after 6 months of RAI		

	Hyperthyroidism No. (%)	Euthyroidism No. (%)	Hypothyroidism No. (%)
Hyperthyroidism	14 (50)	9 (32.1)	5 (17.9)
Euthyroidism	4 (13.3)	19 (63.3)	7 (23.3)
Hypothyroidism	4 (13)	6 (19.3)	21 (67.7)

Overall p-value of the Table is < 0.001 (Fischer Exact Test)

## DISCUSSION

The analyses of our data revealed that hyperthyroidism was cured in majority of our patients, with 38.2% being rendered as Euthyroid, 37% became hypothyroid and 24.7% remained hyperthyroid. The dose of RAI used was in the range from 10 to 30mCi. The literature is widely diverse with some studies showing 100% cure rate to 100% failure rate for hyperthyroidism. (Table-V) Meta-analysis of these studies showed that the difference of outcome depends not only on the RAI dose but also on multiple factors like size of toxic adenoma, radioactive iodine uptake, hyperthyroidism status prior to RAI therapy and variability between patients. We used RAI dose from 10 to 30mCi and our results revealed that although higher doses are associated with more hypothyroidism but it was not consistent and we noticed hyperthyroidism after 6 months even with 25mCi dose of RAI. It clearly reflects that it is not only the dose of RAI that predict the outcome but other factors as mentioned above that effect the individual.

**Table-V:** Review of literature for RAI on toxic nodular goiter.

Author	Number of Patients	RAI dose used in mCi	Outcome		Mean year of follow up	Total years of follow up

			Hyperthyroidism (%)	Hypothyroidism (%)		
Blum M et al., 1975^13^	14	10-15	100	-	6	
Fontana B et al.1980^14^	29	10-40	52	17	10	20
Eyre-Brook & Talbot, 1982^15^	37	12-15	32	5.4	6.5	
Goldstein & Hart, 1983^16^	23	15-55	-	36	8.5	4-16.5
Ross DS et al., 1984^17^	45	5-15	13.3	-	4.9±3.2	0.5-13.5
Hagedus L et al., 1986^18^	27	7.5	7	-	1	
Mariotti S et al., 1986^19^	138	12.6±4.1	15	4	3.2±2.2	1-11
Ratcliffe GE et al., 1986^20^	48	10-15	15	-	3.08	2-10
Huysmans DA et al., 1991^21^	52	20	2	6	10±4	4-17.5
O.Brien T et al., 1992^22^	23	19.7-100	8.7	3.5	3.8	0.4-16.3
Tzavara I et al., 2002^11^	126	25-40	0	5.5	5.3±0.4	1-21
Younis J et al., 2015^23^	60	20-29	0	13.3	5	4-6
Present Study	89	15-20	25.3	36.2		0.5-7

Factors that might have led to these results were prior anti-thyroid drug treatment because less hyper toxic nodule has less susceptibility towards the RAI dose. Toxic nodule usually suppress the normal thyroid tissue and as they are active, it subsequently results in uptake of nearly all the RAI by the toxic nodule while the normal thyroid gland is saved.^11^ Thus complete normalization of thyroid function test prior to RAI may lead to higher incidence of developing hypothyroidism because of fading of this protective mechanism and it might have affected our results as well. It can also lead to incomplete destruction of toxic nodule that can result in persistence of hypethryoid state as one of our patient experienced. Large follicles also lead to the phenomenon of radio resistance and it is due to the fact that large nodule with abundant colloid reduces the effect of beta radiations of RAI.^12^

Our study is in accordance to other studies that failed to show any association of age with RAI outcome.^16^ Although the subgroup analysis showed that the young patients up to the age of 40 years have increased propensity to remain hyperthyroid but the results were not significant. Gender also did not show any significant difference either. None of our patient developed any malignancy in the post RAI period and it is in agreement with the previous studies^24^ but the shorter follow up of our study precludes us from making any significant conclusion.

The major reason for the use of RAI now a days instead of surgery is due to the fact that it is generally considered a very safe procedure and the malignant potential of toxic thyroid nodule is very less.^25^ The only side effect noted with RAI is the development of hypothyroidism that is usually permanent and need lifelong treatment. Previous long term studies have showed that if a patient does not develop hypothyroidism in first year, the chances of development in the subsequent years is very less.^11^

We also compared post RAI thyroid function tests after three and six months and the results showed that the 3^rd^ month TFTs do mirror the true picture with accuracy (p-value < 0.001) although the guidelines^10^ still recommends 6 months TFTs test as the true reflection of the RAI outcome.

## CONCLUSION

In conclusion our study showed that RAI for toxic nodular goiter is safe and very effective treatment. It resolves hyperthyroidism in majority of patients.

## References

[1] Davis AB Toxic Nodular Goiter.

[2] Palos-Paz F, Perez-Guerra O, Cameselle-Teijeiro J, Rueda-Chimeno C, Barreiro-Morandeira F, Lado-Abeal J (2008). Prevalence of mutations in TSHR, GNAS, PRKAR1A and RAS genes in a large series of toxic thyroid adenomas from Galicia, an iodine-deficient area in NW Spain. Eur J Endocrinol.

[3] Van Soestbergen M, Van der Vijver J, Graafland A (1992). Recurrence of hyperthyroidism in multinodular goiter after long-term drug therapy:a comparison with Graves'disease. J Endocrinol Invest.

[4] Bahn RS, Burch HB, Cooper DS, Garber JR, Greenlee MC, Klein I (2011). Hyperthyroidism and other causes of thyrotoxicosis:Management guidelines of the American Thyroid Association and American Association of Clinical Endocrinologists. Thyroid.

[5] Hegedus L, Bonnema SJ, Bennedbæk FN (2003). Management of simple nodular goiter:current status and future perspectives. Endocr Rev.

[6] Bonnema SJ, Hegedus L (2012). Radioiodine therapy in benign thyroid diseases:effects, side effects, and factors affecting therapeutic outcome. Endocr Rev.

[7] Zingrillo M, Urbano N, Suriano V, Modoni S (2007). Radioiodine treatment of Plummer and multinodular toxic and nontoxic goiter disease by the first approximation dosimetry method. Cancer Biother Radiopharm.

[8] Erkan ME, Demirin H, Asik M, Celbek G, Yıldirim M, Aydin Y (2012). Efficiency of radioactive I-131 therapy in geriatric patients with toxic nodular goiter. Aging Clin Exp Res.

[9] Bonnema SJ, Hegedius L (2009). A 30-year perspective on radioiodine therapy of benign nontoxic multinodular goiter. Curr Opin Endocrinol Diabetes Obes.

[10] Ross DS, Burch HB, Cooper DS, Greenlee MC, Laurberg P, Maia AL (2016). 2016 American thyroid association guidelines for diagnosis and management of hyperthyroidism and other causes of thyrotoxicosis. Thyroid.

[11] Tzavara I, Tzanela M, Vlassopoulou B, Kouyioumoutzakis G, Alevizaki C, Thalassinos NC (2002). Long term thyroid function after 1 3 1I treatment for toxic adenoma. HORMONES-ATHENS-.

[12] Miller JM (1975). Plummer's disease. Med Clin North Am.

[13] Blum M, Shenkman L, Hollander CS (1975). The autonomous nodule of the thyroid:correlation of patient age, nodule size and functional status. Am J Med Sci.

[14] Fontana B, Curti G, Biggi A, Fresco G (1980). The incidence of hypothyroidism after radioactive iodine (131I) therapy for autonomous hyper functioning thyroid nodule evaluated by means of life-table method. J Nucl Med Allied Sci.

[15] Eyre-Brook I, Talbot C (1982). The treatment of autonomous functioning thyroid nodules. Br J Surg.

[16] Goldstein R, Hart IR (1983). Follow-up of solitary autonomous thyroid nodules treated with 131I. N Engl J Med.

[17] Ross DS, Ridgway EC, Daniels GH (1984). Successful treatment of solitary toxic thyroid nodules with relatively low-dose iodine-131, with low prevalence of hypothyroidism. Ann Intern Med.

[18] Hegedus L, Veiergang D, Karstrup S, Hansen JM (1986). Compensated 131I-therapy of solitary autonomous thyroid nodules:effect on thyroid size and early hypothyroidism. Acta Endocrinol (Copenh).

[19] Mariotti S, Martino E, Francesconi M, Ceccarelli C, Grasso L, Lippi F (1986). Serum thyroid autoantibodies as a risk factor for development of hypopthyroidism after radioactive iodine therapy for single thyroid'hot'nodule. Acta Endocrinol (Copenh).

[20] Ratcliffe GE, Cooke S, Fogelman I, Maisey MN (1986). Radioiodine treatment of solitary functioning thyroid nodules. Br J Radiol.

[21] Huysmans DA, Corstens FH, Kloppenborg PW (1991). Long-term follow-up in toxic solitary autonomous thyroid nodules treated with radioactive iodine. J Nucl Med.

[22] O'Brien T, Gharib H, Suman VJ, van Heerden JA (1992). Treatment of toxic solitary thyroid nodules:surgery versus radioactive iodine. Surgery.

[23] Younis J, Hussein S (2015). Role and Outcome of RA131 I Therapy in Patients with Autonomous Toxic Adenoma. Egyptian J Nucl Med.

[24] Ryodi E, Metso S, Jaatinen P, Huhtala H, Saaristo R, Valimaki M (2015). Cancer incidence and mortality in patients treated either with RAI or thyroidectomy for hyperthyroidism. J Clin Endocrinol Metab.

[25] Djekidel M, Cai G, Ahmed R, Theoharis C (2014). Correlation of Hot Nodules and Cytopathology:Nine Years at an Academic Institution. OMICS J Radiol.

